# Mental Depression

**Published:** 1888-09-29

**Authors:** 


### The Doctor's Pulpit.

### MENTAL DEPRESSION.

Almost any man who has suffered seriously and often from mental depression would give the whole world his recipe for a cure if he had one. Even we ourselves, who have no faith whatever in self-medication, would depart from our established rule, and defy both etiquette and professional interest, if we knew of any specific which would not fail. This condition has had a great variety of names, and has been the familiar companion of men of all characters and nations. The "melancholy" of our fathers is the "mental depression" of ourselves. Milton, who sometimes loved a pensive mood, knew and hated "loathéd melancholy" of "blackest midnight born." Cromwell, as we know, used to have such profound fits of depression that it was sometimes doubtful whether he was mad or sane. The well-known incident of Luther's conflict with the Devil, when he finally routed the fiend by shying his inkstand at his head, was the close of a long period of solitude and nervous exhaustion. St. Simeon on his pillar divided his time almost equally between a heaven of rapture and a hell of despair. Men of solitude and study know every feature and characteristic of their ghostly companion, melancholy; and men of action, though less familiar with it, yet have their seasons when the spectre holds full sway, and refuses to move an inch at their bidding. Women, perhaps, suffer less frequently than men from this distemper, and it must be admitted they also bear it less well. Many a woman takes to stimulants for no other reason than that she finds the depression of her spirits to be intolerable.

Our fathers, who were more psychological than we, used to attribute the condition to spiritual causes. Among the Puritans a depressed and discontented feeling was regarded as a species of spiritual infirmity, to be struggled against, prayed against, and got rid of by every possible means. It would be easy to raise the laugh against those earnest religionists by pointing out that four or five grains of blue pill would in many instances have been more efficacious in exorcising the devil than many leagues of prayer and psalm-singing. But that would be as unphilosophical on our part as the failure to recognise the occasional hepatic origin of their spiritual deficiencies was on theirs, and with much less justification. We must not blame the Puritan because he was not familiar with the vagaries of his own liver, but the medical men of the times who often knew as little about such things as the patient himself.

Mental depression is undoubtedly in many cases purely physical in origin. On the other hand it is often as distinctly mental. Of purely physical dedepression, one of the commonest of all causes is what is called "derangement of the liver." Some centuries have been known for certain distinguishing characteristics which were of such marked prominence as to make them then and always impossible to be overlooked. Certain periods, for example, have been called "ages of faith," certain others "sceptical epochs," and so on. The latter part of the nineteenth century will probably be signalised by posterity as the "epoch of disordered livers." The liver is "too much with us late and soon." Do we go out to dine? We must have a care for the liver. Do we give up our usual walk in order to finish "Robert Elsmere"? Our liver will make us regret it to-morrow morning. Is the east wind blowing? We must put on a flannel pad to protect the liver. Have we got a headache and a bad temper? We must take some æsculap or sulphate of soda for the liver. Has Mr. Smith refused his wife two guineas for a new bonnet? His liver is out of order, and she thinks of pil. hydrarg. Does Mrs. Smith declare the cook to be a wasteful creature, and the kitchenmaid a saucy slut? She, too, is suffering from disordered liver, and must see Dr. Suaviter, and have an effervescing mixture without delay.

Of course the liver in many or all of these instances is quite as much a vicarious as an actual criminal. In addition to its own share of responsibility for the depression of spirits, it has to stand sponsor for an overloaded stomach, a feeble digestion, an inactive bowel, a general want of tone, and a thousand other causes of malaise. There is no doubt, however, that the condition popularly know as disordered liver gives rise to mental depressions and depths of misery which it is impossible adequately to describe in words.

For this particular kind of melancholy what is right to be done? Perhaps the question may be best answered by stating first what is right not to be done. On no consideration should a person ignorant of medicine undertake a course of medical treatment on his own account. A doctor should be consulted. Even a stupid doctor can do more for the patient than he can do for himself. But there is no need whatever to consult either a stupid or a knavish physician. There are swarms of medical men, both in London and the country, who are perfectly familiar with this kind of trouble, and entirely competent to deal with it. As a matter of fact, the difficulty does not generally lie in the doctor's incompetency, but in the patient's unwillingness to do as he is told. More than once in these pages the doctor's patients have been likened to the clergyman's hearers. The congregation will hear what the parson says, and approve. But they will not carry it out in action, except, of course, a very small minority of them. Similarly the doctor's patients will sit and hear with apparent conviction all that the doctor has got to say; and just as the congregation will attend to the ritual, so will the patient observe what may be called the "ritual" of medical treatment; that is to say, he will take the physic prescribed. But when it comes to the systematic and persistent carrying out of the doctor's directions as to work, diet, rest, exercise, and so on, the patient in most cases completely fails, with the result that the cure is not effected. How exceedingly foolish a man must be who pays a doctor from five shillings to two guineas for his counsel and then goes and deliberately forgets all about it?

We have left ourselves but little space in which to treat of the mental causes of depressed spirits. Worry is one of the most frequent and most powerful of these causes. Any kind of grievance or annoyance which frets the spirit: An unsympathetic husband, an incompetent wife; a rich and not very friendly neighbour; an unmanageable child, a stupid servant; a too small balance at the bank, a too large balance; a dinner party to which you are *not* invited, a dinner party to which you *are* invited; disappointed ambition, too successful ambition—all these may rob the spirit of that easy calm and sober contentment which are essential to perfect health. Mental depression is often really the result of want of brains. A man or a woman has not intelligence enough to take a just view of the situation, and to be content with the lot assigned by circumstances. There is no reason whatever why a man should not try to improve his position and means. But let him do it calmly, patiently, hopefully, intelligently. Do not let him rush at life like a mad bull at a closed gate, and resolve to be through or over at all risks. Any blockhead can treat life that way. The man of intelligence, capacity, and conviction can wait as well as work; and if success equals his expectation he will be pleased, but not delirious with pride and joy. If on the other hand the "sequestered vale of life" be now and always his portion, he is able to take the just measure of all wordly successes, and to be satisfied and pleased with his own modest share. The temper of mind inculcated by a reasonable Christianity, and by an telligent Judaism as well, is of great value as a medicinal agent. In many cases it would be of more service than all the drugs in the pharmacopœia, and all the physicians of all the Royal Colleges. It would be a great gain to all people who suffer from melancholy and depression if they clearly knew how very much they could do to help themselves, and, above all, if knowing this, they would thoroughly and steadfastly carry it out in practice.

